# Evaluating health information provided to kratom consumers by good manufacturing practice-qualified vendors

**DOI:** 10.1186/s13011-023-00531-4

**Published:** 2023-04-11

**Authors:** Katherine Hill, Stephen Gibson, Oliver Grundmann, Kirsten E. Smith, Jonathan Ballard, Corneliu N. Stanciu

**Affiliations:** 1 Department of Epidemiology of Microbial Diseases, Yale School of Public Health, New Haven, CT USA; 2 Massachusetts College of Pharmacy and Health Sciences, Manchester, NH USA; 3 New Hampshire Hospital, Concord, NH USA; 4 College of Pharmacy, Department of Medicinal Chemistry, University of Florida, Gainesville, FL USA; 5 College of Pharmacy, Department of Pharmaceutical Sciences, Midwestern University, Glendale, AZ USA; 6 National Institute On Drug Abuse Intramural Research Program, Baltimore, MD USA; 7 New Hampshire Department of Health and Human Services, Concord, NH USA; 8 Department of Psychiatry, Dartmouth-Hitchcock Medical Center, Lebanon, NH USA; 9 Geisel School of Medicine, Dartmouth College, Hanover, NH USA

**Keywords:** Kratom, DISCERN, Herbal, *Mitragyna speciosa*, Healthcare, Website evaluation

## Abstract

**Background:**

“Kratom” commonly refers to the botanical *Mitragyna speciosa*, native to Southeast Asia, which is increasingly used globally for its unique pharmacological effects. Motives for using the whole plant material or kratom-derived products include self-management of pain, mental health disorders, symptoms related to substance use disorders, and/or to increase energy. In the United States, kratom products have varying alkaloid content, potencies, and marketing profiles. There is little regulatory oversight over kratom, as it is currently not approved as a dietary supplement by the Food and Drug Administration. This results in substantial variability in labeling of kratom products and the product information provided to consumers.

**Methods:**

In January 2023, we evaluated the American Kratom Association’s Good Manufacturing Practices (GMP) qualified vendors’ websites (*n* = 42) using the well-established and validated DISCERN instrument to examine the quality of health information provided to consumers. DISCERN contains 15 five-point Likert-scale questions on specific criteria, with the highest possible score being 75, indicating that all the DISCERN criteria have been fulfilled by the website (i.e., the highest quality information is provided to consumers).

**Results:**

The mean DISCERN score for all evaluated online kratom vendors was 32.72 (SD = 6.69; score range 18.00–43.76). Overall, vendors scored higher on DISCERN questions assessing the website’s reliability, as vendors typically provided clear information for consumers about product availability, purchasing, shipping, etc. On average, vendors scored poorly on the DISCERN section pertaining to the quality of the health information provided. Information on kratom’s potential risks and benefits was particularly insufficient.

**Conclusions:**

Consumers require high quality information in order to make informed decisions concerning use, which entails disclosure of known risks and potential benefits. The online kratom vendors evaluated in this study should consider enhancing the quality of health information provided, especially information regarding kratom’s risks and benefits. Further, consumers should be made aware of current knowledge gaps related to kratom’s effects. Clinicians must also be aware of the lack of evidence-based information available to their patients who use kratom or are interested in using kratom products, in order to facilitate educational discussions with them.

## Background

In recent years there has been a steady increase in interest, importation, and use of kratom (*Mitragyna speciosa* Korth.) leaf products in the United States (US) [1, 2]. Various epidemiological studies estimate the lifetime kratom use among the adult American population to range between 0.9 and 6.1% [3, 4]. Motivations for use include “self-management” of symptoms related to chronic pain, fatigue, substance use disorders, and psychiatric conditions along with attenuating opioid-withdrawal [1, 5–7]. Use of kratom as a short- or long-term substitute for opioids, stimulants, and alcohol has also been described [2, 8]. Despite the numerous perceived therapeutic benefits reported by those who use kratom, there are no clinical safety/tolerability studies, human abuse potential studies, or randomized clinical trials investigating kratom’s risks or efficacy for any clinical indication [9, 10]. Moreover, the current state of the literature does not permit any robust, or generalizable, conclusions to be drawn concerning kratom’s role as a harm reduction tool [11].

Kratom has a long history of use in its indigenous habitat of Southeast Asia, where the majority of consumption occurs via chewing or brewing of the leaves as a tea, and to a lesser extent smoking. Unlike the traditional modes of kratom use, unregulated products marketed in the US are diverse in terms of alkaloid content and composition (rather than the varied, but natural alkaloid composition in fresh leaves), and hence differ in potency [12]. Dosing and product formulation also differs in that many products are not fresh, but rather derived from dried leaves in the form of raw powder, capsules, and concentrated extracts formulated into tablets, liquids, and other preparations. These products are readily available for purchase in gas stations, specialized convenience stores, and from online vendors. One study performed during the COVID-19 pandemic found that 72.7% of people purchased kratom online [13].

Contrasting the benefits reported by consumers, some state and local governments as well as the US Food and Drug Administration (FDA) are concerned about potential harms associated with kratom [12]. These concerns stem primarily from the quality of products available in the US, which have unknown purity (e.g., potential adulterants and contaminants) and the potential harms resulting from co-ingestions—specifically alkaloid interactions with prescribed pharmaceuticals, illicit substances, over-the-counter medications, and other products marketed as supplements [14]. As such, kratom is currently unregulated in the US and *not* approved by the FDA as a dietary supplement. Thus, the sale of kratom products has little regulatory oversight and consumers have been largely uninformed about what they are actually taking when using a “kratom product” [15].

However, the American Kratom Association (AKA) is an industry-associated advocacy group that lobbies for consumers’ right to use kratom. They have been working at the state-level to reverse or prevent prohibitions, and to introduce Kratom Consumer Protection Act (KCPA) legislation for state-level regulation of commercial kratom sales. Through this act, kratom regulations get introduced on age restrictions, product labeling, lab testing requirements, fines, and the sale of kratom. Some states, such as Alabama, Rhode Island, and Vermont, have statewide kratom bans, while other states have specific counties, municipalities, or cities with kratom bans, such as San Diego, California [16]. To address these bans, and prevent future ones, the AKA lobbies for policy makers to adopt state specific versions of a KCPA [17].

In addition to this, the AKA has recently undertaken efforts to provide guidance for improved quality of products. One such endeavor comes through the Good Manufacturing Practices (GMP) Standards Program, which are guidelines for vendors and manufacturers to follow [18]. Vendors registered with the AKA must adhere to all GMP regulations and pass a third-party audit. These requirements pertain to standard operating procedures, record keeping, adverse event reporting systems, product labeling, product recalls, market practice, and state regulations. Maintenance of registration also requires annual audit. This process may ensure some quality control over the products provided to consumers from GMP-certified vendors, but it is not a guarantee. Most who use kratom regularly (daily or > 4 times weekly) appear to purchase primarily online, even with some in-person purchasing or changes in vendors over time [2, 19]. Consumers purchasing online must ultimately make decisions about available kratom products based on the information available through online sources, including vendors who should be best positioned to describe the exact products they are selling. However, avenues for accessing credible, data-driven information in a manner which the average consumer can understand remain limited.

A 2021 study by Ng et al. evaluated the quality of consumer health information available on the websites of 51 kratom vendors who sold products in Canada. The authors concluded that kratom consumers were not provided with critical information needed for informed decision-making concerning use, particularly of the risks and the impact on their health [15]. Indeed, 40 of the evaluated sites were rated by the authors as having a “poor” quality of information. Here, we utilized the same assessment methods, but instead focused only on vendors registered with the AKA’s GMP program.

## Methods

### Eligibility criteria

Eligible vendor websites were selected based on the AKA’s list of GMP-qualified vendors as of January 19, 2023 [18]. Only vendors who had an active website domain with information about their kratom product(s) were included in our analysis. Thus, one vendor, Kratom Kaps, was excluded from our analysis.

### Data extraction and website quality assessment

All eligible vendor websites were examined by K.H. and the following information was ascertained: URL, kratom products sold, non-kratom products sold, the presence of a disclaimer, and whether the site required age confirmation for entry. Accuracy of the information extracted was confirmed by multiple authors.

The DISCERN instrument, a validated tool for the evaluation of written consumer health information, was devised in the late 1990’s by a multidisciplinary team in the United Kingdom [20]. DISCERN has been used to evaluate written health information in print and online [21]. The instrument contains 16 questions, designed to assess whether consumers are provided quality health information that is supported by relevant evidence [22]. The first 15 questions of DISCERN represent individual criteria related to a specific aspect of the quality of the written consumer health information, while the final question represents a global score of the publication. Section 1 (questions 1–8) permits a thorough examination of the vendor’s website reliability using questions regarding its aims, relevancy, sources of information or support, and potential bias. Section 2 (questions 9–15) evaluates the quality of the consumer health information on topics such as descriptions of treatment mechanism, risks, and benefits. Section 3 contains the final evaluation (question 16), which evaluates shortcomings. All questions of the DISCERN instrument take the form of a five-point Likert scale, with the highest possible score of 75. Higher DISCERN scores indicate that the health information provided to consumers is of greater reliability and quality. To assure that evaluators use DISCERN consistently, the instrument provides what the DISCERN creators call “hints” for each question, which prompt the evaluator to look for and assess certain key indicators for each question’s given criteria.

In order to compare our current study findings to the previous study completed by Ng et al., we followed their methods with few modifications [15]. As such, K.H., C.S., and S.G. individually piloted the DISCERN instrument on three websites and met to discuss their scoring methods to help standardize application of DISCERN. After discussion of discrepancies, K.H., C.S., and S.G. independently applied the DISCERN instrument over a three-day period (January 20 to January 22, 2023) to ensure websites would not change substantially during active evaluation. K.H., C.S., and S.G. then met to discuss and reconcile any differences > 2 points on the Likert scale (e.g., one author evaluated the website as a 1 for question 4 while another author evaluated it as a 4). After differences were reviewed, all three evaluators’ scores for questions 1 through 15 were averaged and summed, to calculate a DISCERN score between 15 and 75. Standard deviations were calculated for each question and the overall DISCERN score. All analyses were conducted in Microsoft Excel.

## Results

### General characteristics of eligible websites

A total of 42 eligible websites were evaluated (Table 1). Across vendors, there were a variety of kratom products sold, including powder, capsules, tea, gummies, shots, chewable tablets, and more. Some websites (17/42) sold other products such as kava, cannabidiol, apparel, and digital scales, while the majority of vendors exclusively sold kratom products. Most websites (38/42) contained some form of disclaimer about kratom consumption on the website, often in a separate tab or at the bottom of the web page. Only 9 out of 42 vendors prompted consumers to confirm their age prior to entering the website, with 18 years of age being the most common age for entry (8/42).

**Table 1:** Characteristics of eligible AKA GMP qualified vendors

Company Name	URL	Kratom Products	Other Products	Disclaimer	Age for Entry
1836 Kratom	https://www.1836kratom.com/	Powdered tea leaf, capsules, liquid extracts	None	Yes	No
Austin Organic Village	https://austinorganicvillage.online/	Powder, capsules, extracts, blends, leaves	None	Yes	Yes, 21 +
Authentic Kratom	https://www.authentickratom.com/	Crushed leaf, powder, extract, capsules	Face mask, Incense	Yes	Yes, 18 +
Buy Kratom Bulk USA	https://buykratombulkusa.com/	Powder, capsules	None	Yes	No
Chief Kratom	https://chiefkratom.com/	Extracts	None	Yes	No
Choice Botanicals	https://choicekratom.com/	Powder, capsules, extracts	THC, rolling paper	No	Yes, 18 +
Christopher's Organic Botanicals	https://christophersorganicbotanicals.com/	Tea powder, tea, capsules, crushed leaf, extracts	None	Yes	No
Crisp Kratom	https://crispkratom.com/	Gummies, capsules, powder	None	Yes	Yes, 18 +
First Coast Tea	https://www.firstcoastteaco.com/	Tea	Essential oils, kava, herbal soaps, coffee, teas, botanicals, apparel, posters, edible film pouches	No	No
Golden Rule Botanicals	https://goldenrulebotanicals.com/	Tea, blends	CBD, shilajit, kava, subscription boxes, teas, apparel, cups, edible film pouches	Yes	No
Happy Hippo	https://happyhippo.com/	Extracts, capsules, powder, taffy chews	Apparel, cups	Yes	No
Harvest Kratom	https://harvestkratom.com/	Extracts, gummies, powder, capsules	None	Yes	No
Hush Kratom	https://hushkratom.com/	Extracts, gummies, softgels, capsules, enhanced powder	None	Yes	No
K Chill	https://www.k-chill.com/	Powder, instant coffee, capsules, extract capsules, extract shots, tinctures	None	Yes	No
Kats Botanicals	https://katsbotanicals.com/	Powder, capsules, extracts, blends, gummies	Guidebook	Yes	No
King Kratom	https://kingkratom.com/	Extracts, powder, capsules	None	Yes	No
Kosta Kratom	https://www.kostakratom.com/	Powder, shots, capsules	None	Yes	No
Kraken Kratom	https://krakenkratom.com/	Powder, blends, leaf, tea bags, capsules, shots, honey sticks, chewable tea tablets, gummies, extracts, enhanced powders, softgels	Kava, empty capsules, digital scales, cups, apparel, tea accessories, book	No	No
Kratom Divine	https://kratomdivine.com/	Powder, capsules, liquids	None	Yes	No
Kratom krates	https://kratomkrates.com/	Powder, capsules, extracts, gummies	None	Yes	No
Kratom Now	https://kratomnow.com/	Powder, gummies, extracts	None	Yes	Yes, 18 +
Kratom Source USA	https://kratomsourceusa.com/	Powder, capsules	None	Yes	No
Kratom Spot	https://kratomspot.com/	Powder, leaves, capsules, extracts, shots	Empty capsules, capsule filling machine, tea accessories, apparel	Yes	No
Kryptic Kratom	http://kryptickratom.com/	Shots, tinctures, extract capsules	None	Yes	No
Laughing Lion Herbs	https://www.laughinglionherbs.com/	Shots, powder, capsules	Kava	Yes	No
Left Coast Kratom	https://left-coast-kratom.com/	Capsules, powder, gummies, extracts, shots	Tea accessories, hand sanitizer, bottle, digital scales, empty capsules, book	No	No
MIT 45	https://mit45.com/	Extracts, capsules, powdered leaf	None	Yes	No
New Dawn Kratom	https://newdawnkratom.com/	Powder	None	Yes	No
NuWave Botanicals	https://nuwavebotanicals.com/	Powder, capsules, chewable tablets, shots, soda	None	Yes	Yes, 18 +
Oasis Kratom Products	https://oasiskratom.com/	Powder, gummies, capsules, crushed leaf, extracts	Kava, botanicals	Yes	No
Optimized Plant Mediated Solutions	https://opmkratom.com/	Capsules, extracts	Kava	Yes	No
Organic Kratom USA	https://organickratomusa.com/	Powder, blends, capsules, extracts	CBD	Yes	No
Phytoextractum	https://www.phytoextractum.com/	Capsules, extracts, chewable tablets, crushed leaf, powder	Mushrooms, kava, CBD, kombucha, maca, botanicals, digital scales, essential oils, empty capsules, coffee, books, smudge sticks, tumeric, tea, hand sanitizer	Yes	No
Premium EXP Kratom	https://enjoyexperiencekratom.com/about-us/	Powder, shots, tabs, capsules, E gel	None	Yes	No
PurKratom	https://www.purkratom.com/	Powder, capsules, shots, gummies	Ginger and tumeric gummies, tumeric capsules	Yes	Yes, 21 +
Remarkable Herbs	https://remarkableherbs.com/	Powder	Kava, specialty herbs	Yes	No
SK	https://soapkorner.com/	Powder, capsules, blends, shots	Hemp, kava, CBD, MCT oil	Yes	No
Super Speciosa Raw Leaf	https://superspeciosa.com/	Powder, capsules, tea bags, chewable tablets, gummies	None	Yes	No
The Golden Monk	https://goldenmonk.com/	Capsules, powder	None	Yes	No
Tusk Ultra Premium Kratom	https://tuskkratom.com/	Powder, extracts, liquids, capsules, gummies	None	Yes	Yes, 18 +
Viable Solutions	https://viablekratom.com/	Capsules, powder, extracts, liquids, tinctures	None	Yes	Yes, 18 +
Whole Herbs	https://wholeherbskratom.com/	Powder, capsules	None	Yes	No

### DISCERN instrument ratings: total score

Table 2 presents the mean scores for each website across raters following the DISCERN criteria, alongside the standard deviation of scores between the researchers and across the websites for each criterion. The overall DISCERN score for each site is also provided. The highest DISCERN score was 43.76 and the lowest was 18.00, whereas the total mean score was 32.72 (SD = 6.69) out of a maximum of 75.00.

**Table 2:** DISCERN instrument ratings

		**SECTION 1: Is the publication reliable?**							
**Vendor**	**Website**	**1. Are the aims clear?**	**2. Does it achieve its aims?**	**3. Is it relevant?**	**4. Is it clear what sources of information were used to compile the publication (other than the author or producer)?**	**5. Is it clear when the information used or reported in the publication was produced?**	**6. Is it balanced and unbiased?**	**7. Does it provide details of additional sources of support and information?**	**8. Does it refer to areas of uncertainty?**
1836 Kratom	https://www.1836kratom.com/	4.67	4.67	3.67	2.00	1.67	3.67	3.33	3.00
Austin Organic Village	https://austinorganicvillage.online/	4.67	4.67	3.00	3.67	2.67	2.00	3.00	2.00
Authentic Kratom	https://www.authentickratom.com/	4.33	4.33	4.33	2.00	2.00	2.00	2.67	1.67
Buy Kratom Bulk USA	https://buykratombulkusa.com/	3.67	3.67	2.67	1.67	2.00	1.33	2.33	1.67
Chief Kratom	https://chiefkratom.com/	2.67	2.67	2.00	1.00	1.00	1.33	1.67	2.67
Choice Botanicals	https://choicekratom.com/	3.67	3.67	2.00	1.00	2.00	1.00	1.33	1.00
Christopher's Organic Botanicals	https://christophersorganicbotanicals.com/	4.67	4.67	3.00	2.00	1.67	1.67	2.33	2.00
Crisp Kratom	https://crispkratom.com/	3.00	2.00	2.00	1.67	1.67	1.67	1.67	2.00
First Coast Tea	https://www.firstcoastteaco.com/	2.00	1.67	1.00	1.00	1.00	2.00	1.33	1.00
Golden Rule Botanicals	https://goldenrulebotanicals.com/	3.33	3.00	1.67	1.33	1.33	1.67	1.00	1.67
Happy Hippo	https://happyhippo.com/	4.67	4.67	3.67	1.33	2.00	2.00	2.33	1.67
Harvest Kratom	https://harvestkratom.com/	4.00	3.67	1.67	1.00	1.00	1.67	1.00	1.33
Hush Kratom	https://hushkratom.com/	3.33	3.00	1.67	1.33	1.00	1.67	1.00	1.67
K Chill	https://www.k-chill.com/	3.33	3.33	2.00	1.67	1.67	2.00	2.33	1.33
Kats Botanicals	https://katsbotanicals.com/	4.67	4.67	3.33	2.00	2.00	2.00	2.33	1.67
King Kratom	https://kingkratom.com/	3.00	3.00	2.33	1.33	1.00	1.00	1.00	2.00
Kosta Kratom	https://www.kostakratom.com/	3.33	3.00	1.67	1.00	1.00	1.33	1.33	1.67
Kraken Kratom	https://krakenkratom.com/	4.33	4.33	3.33	2.00	1.67	2.67	2.33	3.67
Kratom Divine	https://kratomdivine.com/	4.67	4.33	3.67	2.00	1.67	1.67	2.67	3.00
Kratom kaps	-	-	-	-	-	-	-	-	-
Kratom krates	https://kratomkrates.com/	3.67	3.67	1.67	1.00	1.00	1.67	1.33	2.00
Kratom Now	https://kratomnow.com/	4.67	4.67	2.67	1.67	1.33	2.67	2.33	2.00
Kratom Source USA	https://kratomsourceusa.com/	4.00	4.00	2.67	1.33	1.33	1.67	2.00	1.67
Kratom Spot	https://kratomspot.com/	4.67	4.33	3.00	2.00	2.00	2.33	2.67	2.00
Kryptic Kratom	http://kryptickratom.com/	3.00	3.00	1.67	1.33	1.33	1.33	1.00	1.33
Laughing Lion Herbs	https://www.laughinglionherbs.com/	4.67	4.33	4.33	2.00	1.67	3.00	2.00	3.00
Left Coast Kratom	https://left-coast-kratom.com/	4.00	4.00	2.67	2.33	2.00	2.33	2.00	1.33
MIT 45	https://mit45.com/	3.67	3.67	3.00	1.33	1.67	2.00	2.00	1.67
New Dawn Kratom	https://newdawnkratom.com/	4.33	4.33	3.33	3.00	2.33	3.00	3.33	3.00
NuWave Botanicals	https://nuwavebotanicals.com/	3.67	3.33	2.67	1.33	1.67	2.00	2.00	1.67
Oasis Kratom Products	https://oasiskratom.com/	4.33	4.33	3.00	1.67	2.00	2.33	3.00	3.00
Optimized Plant Mediated Solutions	https://opmkratom.com/	4.33	4.33	3.00	2.00	1.00	2.33	2.00	3.33
Organic Kratom USA	https://organickratomusa.com/	4.00	3.67	2.67	2.00	2.00	1.67	2.67	2.00
Phytoextractum	https://www.phytoextractum.com/	4.00	3.67	2.67	2.00	2.00	2.67	2.67	1.33
Premium EXP Kratom	https://enjoyexperiencekratom.com/about-us/	4.00	4.00	2.33	1.00	1.33	1.33	1.00	1.33
PurKratom	https://www.purkratom.com/	4.67	4.67	4.00	1.67	1.33	2.67	2.00	2.67
Remarkable Herbs	https://remarkableherbs.com/	3.00	2.67	2.67	1.33	1.00	2.00	1.33	2.00
SK	https://soapkorner.com/	4.33	4.33	2.67	1.67	1.67	3.00	2.33	2.33
Super Speciosa Raw Leaf	https://superspeciosa.com/	4.67	4.67	3.33	2.67	2.00	2.33	2.67	2.00
The Golden Monk	https://goldenmonk.com/	4.67	4.67	2.67	1.33	1.00	2.67	2.33	2.00
Tusk Ultra Premium Kratom	https://tuskkratom.com/	4.33	4.00	2.67	2.33	2.33	2.33	3.00	2.33
Viable Solutions	https://viablekratom.com/	4.67	4.67	2.67	4.33	3.00	2.33	3.33	1.67
Whole Herbs	https://wholeherbskratom.com/	4.00	4.00	3.33	1.33	2.00	2.33	1.67	2.67
**Total Mean Score**		**3.98**	**3.86**	**2.71**	**1.75**	**1.64**	**2.06**	**2.09**	**2.02**
**Total Standard Deviation**		**0.67**	**0.77**	**0.76**	**0.70**	**0.50**	**0.58**	**0.70**	**0.64**

	**SECTION 2: How good is the quality of information on treatment choices?**							**SECTION 3: Overall rating?**		
**Vendor**	**9. Does it describe how each treatment works?**	**10. Does it describe the benefits of each treatment?**	**11. Does it describe the risks of each treatment?**	**12. Does it describe what would happen if no treatment is used?**	**13. Does it describe how the treatment choices affect overall quality of life?**	**14. Is it clear that there may be more than one possible treatment choice?**	**15. Does it provide support for shared decision-making?**	**16. Based on the answers to all of the above questions, rate the overall quality of the publication as a source of information about treatment choices**	**Standard Deviation of Overall Score (Q16)**	**DISCERN Score (Sum of Q1-Q15)**
1836 Kratom	3.00	3.00	2.33	1.33	2.00	2.00	3.00	3.33	**1.15**	**43.33**
Austin Organic Village	3.33	3.67	2.00	1.00	1.00	1.00	2.67	2.67	**0.58**	**40.33**
Authentic Kratom	3.67	3.00	2.00	1.00	1.67	1.67	2.00	2.67	**0.58**	**38.33**
Buy Kratom Bulk USA	2.00	1.67	1.33	1.00	1.33	1.00	1.33	1.67	**0.58**	**28.67**
Chief Kratom	1.33	1.00	2.33	1.00	1.00	1.00	2.67	2.00	**0.00**	**25.33**
Choice Botanicals	2.33	2.33	1.00	1.00	1.67	1.00	1.33	1.67	**0.58**	**26.33**
Christopher's Organic Botanicals	3.67	3.00	2.00	1.00	1.00	1.33	2.33	2.67	**0.58**	**36.33**
Crisp Kratom	2.00	2.00	1.33	1.00	1.33	1.33	1.33	1.67	**0.58**	**26.00**
First Coast Tea	1.00	1.00	1.00	1.00	1.00	1.00	1.00	1.33	**0.58**	**18.00**
Golden Rule Botanicals	1.00	1.00	1.33	1.00	1.33	1.00	1.67	1.67	**0.58**	**23.33**
Happy Hippo	3.33	3.67	1.33	1.00	1.67	1.33	3.00	3.00	**0.00**	**37.67**
Harvest Kratom	1.67	2.00	1.33	1.00	1.00	1.00	1.33	1.67	**0.58**	**24.67**
Hush Kratom	1.33	1.33	3.67	1.00	1.67	1.00	2.00	2.33	**0.58**	**26.67**
K Chill	1.33	1.67	1.67	1.00	1.67	2.67	1.67	2.00	**1.00**	**29.33**
Kats Botanicals	2.33	3.33	1.67	1.00	1.33	1.00	2.00	2.33	**0.58**	**35.33**
King Kratom	1.67	1.00	2.33	1.00	1.00	1.00	2.67	1.67	**0.58**	**25.33**
Kosta Kratom	1.00	1.00	1.67	1.00	1.00	1.00	1.67	1.33	**0.58**	**22.67**
Kraken Kratom	1.67	1.67	1.67	1.00	1.00	1.67	2.33	2.67	**0.58**	**35.33**
Kratom Divine	3.00	3.00	3.33	1.00	1.67	1.00	3.00	3.33	**1.15**	**39.67**
Kratom kaps	-	-	-	-	-	-	-	-	**-**	**-**
Kratom krates	1.67	1.67	1.33	1.00	1.00	1.00	1.33	1.67	**0.58**	**25.00**
Kratom Now	2.67	3.00	1.67	1.00	1.67	1.00	2.33	2.67	**0.58**	**35.33**
Kratom Source USA	1.67	2.00	1.33	1.00	1.67	1.33	1.33	1.67	**0.58**	**29.00**
Kratom Spot	3.00	3.00	3.00	1.00	1.67	1.67	2.67	3.00	**1.00**	**39.00**
Kryptic Kratom	1.67	2.67	1.00	1.00	1.00	1.00	1.33	1.67	**0.58**	**23.67**
Laughing Lion Herbs	3.00	3.67	3.67	1.00	1.67	2.33	3.33	3.33	**1.15**	**43.67**
Left Coast Kratom	2.00	2.33	1.67	1.33	1.33	1.67	2.33	2.33	**0.58**	**33.33**
MIT 45	2.00	2.33	1.33	1.00	1.33	1.00	1.33	1.67	**0.58**	**29.33**
New Dawn Kratom	2.67	3.33	3.67	1.00	1.67	1.67	2.67	3.33	**0.58**	**43.33**
NuWave Botanicals	3.00	3.67	2.00	1.00	1.67	1.67	2.33	2.33	**0.58**	**33.67**
Oasis Kratom Products	2.67	2.33	1.67	1.00	1.00	1.67	3.00	3.00	**0.00**	**37.00**
Optimized Plant Mediated Solutions	1.33	1.00	3.67	1.00	1.00	1.33	3.00	2.67	**0.58**	**34.67**
Organic Kratom USA	2.33	2.33	1.67	1.00	1.33	1.00	2.67	2.00	**0.00**	**33.00**
Phytoextractum	2.00	2.33	1.00	1.00	1.33	2.33	1.67	2.67	**0.58**	**32.67**
Premium EXP Kratom	1.67	1.67	2.00	1.00	1.33	1.00	1.67	1.67	**0.58**	**26.67**
PurKratom	3.67	3.67	3.00	1.00	1.33	1.67	2.67	3.00	**0.00**	**40.67**
Remarkable Herbs	1.33	1.33	1.67	1.00	1.00	1.00	2.00	1.67	**0.58**	**25.33**
SK	3.00	2.67	2.00	1.00	1.67	1.00	2.33	2.67	**1.15**	**36.00**
Super Speciosa Raw Leaf	3.00	3.00	2.33	1.00	1.67	1.33	2.67	3.00	**1.00**	**39.33**
The Golden Monk	2.33	2.00	2.00	1.00	1.67	1.33	2.67	2.33	**0.58**	**34.33**
Tusk Ultra Premium Kratom	2.67	4.00	3.67	1.00	1.67	1.00	3.67	3.00	**1.00**	**41.00**
Viable Solutions	1.67	2.00	1.67	1.00	1.33	1.00	2.67	2.67	**0.58**	**38.00**
Whole Herbs	3.00	2.67	3.00	1.00	2.00	1.67	3.00	3.00	**1.00**	**37.67**
**Total Mean Score**	**2.25**	**2.36**	**2.03**	**1.02**	**1.39**	**1.33**	**2.23**	**2.35**	**0.61**	**32.72**
**Total Standard Deviation**	**0.79**	**0.89**	**0.82**	**0.07**	**0.31**	**0.43**	**0.67**	**0.62**	**0.31**	**6.69**

### DISCERN instrument ratings: sect. 1

For the specific criteria composing the total score, section 1 of the DISCERN instrument focuses on the reliability of the consumer health information. Question 1 and 2 evaluate the aims and achievement of the aims of the website. Vendor websites who scored a 4 or 5 on these questions generally were easy to navigate, presented their status as a vendor obviously, and provided clear information on the origin and manufacturing of their kratom product(s). Most (64.29%) websites scored a 4 or higher on question 1, with the total mean score of 3.98 out of 5 across all websites (SD = 0.67).

For question 3, regarding relevancy, vendors scored higher on the Likert scale if they provided answers to anticipatory consumer questions. The authors assumed what a kratom novice’s potential questions might be, and whether the site had information to address such unknowns. Specifically, we evaluated information about methods of kratom consumption, when to use kratom, and at what dosage. The mean score was 2.71 out of 5 (SD = 0.76) for question 3.

Questions 4 and 5 evaluate aspects of the information provided to consumers. The majority of websites (85.71%; 36 out of 42) scored 2 or lower on question 4, as they provided little reference to the sources of the information or claims made on their sites. Thus, the total mean score of question 4 was 1.75 (SD = 0.70). Question 5 results were similar across the study sample, with a mean of 1.64 (SD = 0.50). When evaluating whether the websites were balanced or unbiased, the researchers scored websites that presented kratom information in a neutral manner that included both pros and cons of kratom use (e.g., a product may provide the desired workout boost but may also give you a twitchy feeling). However, most websites tended toward providing information that highlighted or emphasized positives of kratom, without equal discussion of risks, resulting in question 6 having a total mean score of 2.06 (SD = 0.58). Similarly, most websites did not provide additional resources nor address the uncertainties of kratom, such as the current gaps in knowledge about possible side effects. As such, the total mean score for question 7 was 2.09 (SD = 0.70) and for question 8 was 2.02 (SD = 0.64).

### DISCERN instrument ratings: sect. 2

Section 2 of the DISCERN instrument allows researchers to examine the quality of health information provided to consumers, starting with evaluating whether the material describes how treatments work. The researchers searched for information regarding kratom’s mechanism and pharmacology. Websites that described how mitragynine and 7-hydroxymitragynine alkaloids act on opioid receptors, or some variation of this in lay terms, tended to score higher [23]. In total, the mean score for question 9 was 2.25 (SD = 0.79).

Questions 10 and 11 evaluate the descriptions of the potential risks and benefits of using the advertised product. In the case of the AKA GMP qualified vendors, the total mean score for question 10 was 2.36 (SD = 0.89), which represents that websites often lacked sufficient information on the benefits of kratom use. Question 11 had a total mean score of 2.03 (SD = 0.82), as many sites did not fully describe or speculate about some of the better-known risks of kratom use. For questions 12, 13, and 14, which evaluate the information on treatment options, the websites tended to score poorly, with mean scores falling just above 1 for all three questions. Ultimately, when evaluating whether or not the vendors provided sufficient information to facilitate shared decision-making between patients and providers (question 15), the total mean score was 2.23 (SD = 0.67), indicating that many websites either did not or only partially provided adequate or high-quality health information to consumers.

### DISCERN instrument ratings: sect. 3 

Regarding the overall rating of the eligible AKA GMP qualified vendors, the total mean score was 2.35 (SD = 0.62). This overall score falls between a 1 (serious or extensive shortcomings) and a 3 (potentially important but not serious shortcomings) on the DISCERN Likert scale for question 16. More than 25% of websites (11/42) received a score of 3 or higher, reflecting they fell between potentially important but not serious shortcomings (i.e., a 3) and minimal shortcomings (i.e., a 5).

## Discussion

In contrast to the previous assessment of 51 online vendors for Canadian kratom consumers, which found an average DISCERN score of 29.61 (SD = 7.34), our study of AKA GMP-qualified vendors found an average DISCERN score of 32.72 (SD = 6.69) [15]. Though our study provides evidence of slightly higher DISCERN scores for the AKA GMP qualified vendors than the online Canadian kratom vendors assessed by Ng et al., it is unclear whether the AKA GMP qualification meaningfully differentiates the two samples of vendor websites. For instance, it might have been reasonable to suspect that GMP-certified vendor websites would have scored higher, on average, compared to previously assessed non-GMP-certified vendor websites using the same instrument. Although GMP-certification through the AKA may promote higher industry standards for internal accountability and self-regulation, this does not seem to translate to improvements in quality public-facing information that consumers need about the kratom products they are purchasing. The GMP-certified “branding” among online vendors may be used to *imply* quality assurance practices to consumers, but such assurances or signals do not substitute for detailed product information.

It is evident from our findings that there is considerable room for improvement, especially when contrasting with online vendors for approved dietary supplements. For instance, previous studies indicate that weight loss agent online vendor websites scored an average of 44.80 utilizing the DISCERN instrument [24], while online vendors selling supplements targeted at improving fatigue symptoms scored an average of 47.64 [25]. While dietary supplement products seemingly struggle to achieve a perfect score using the DISCERN criteria, kratom websites should still seek to improve when it comes to product information and safety. Specifically, online kratom vendors that we assessed performed poorly with respect to criteria in Sects. 2 and 3, which focus on the quality of the health information regarding risks, benefits, and unknown effects provided.

The implications of this exploratory assessment are two-fold. First, it provides guidance to kratom vendors, legislators, and regulatory bodies on elements needed as part of future kratom policy development. Second, it provides physicians and other healthcare professionals with an understanding of the quality of information available, prompting more informed discussions and education for patients who seek to, or actively use, kratom.

As stated earlier, kratom is not a federally, or state, regulated industry which results in variation in what vendors are required to include on their website. While most vendors had some form of disclaimer related to kratom, the exact content of this disclaimer varied (e.g., disclaimers about age restrictions, disclaimers about how the product is not intended to treat a health condition, disclaimers about where kratom is banned, etc.). Currently, for FDA-approved dietary supplements, the Dietary Supplement Health and Education Act (DSHEA) stipulates that vendors can only provide structure–function claims, which describe the intended role of the given supplement. These are typically relatively vague statements, such as a product may improve sleep or may lessen fatigue. Subsequently, DSHEA requires manufacturers to clearly state, in bold, “This statement has not been evaluated by the Food and Drug Administration. This product is not intended to diagnose, treat, cure, or prevent any disease” [26].

However, kratom is not an FDA-approved dietary supplement. Thus, the AKA and kratom vendors in the US are *limited* in what information they can legally present to consumers – even if they wanted to – because of (a) the substantial lack of data on kratom’s effects, and (b) the legal limitations of the DSHEA about what information can be provided. Further, in recent years, the FDA has issued warning letters to kratom vendors who make “fraudulent health claims,” “unsubstantiated claims,” or sell “unapproved kratom products” [27–29]. The FDA continues to monitor kratom vendors and has expressed a desire for consumers to be provided with adequate and accurate information. As such, vendors are unable to promote content or discuss any potential health benefits or risks associated with, or speculated to be associated with, kratom without risk of being reprimanded or fined. While it is imperative that consumers are not sold products under fraudulent or unsubstantiated circumstances, there is very little that vendors can include on their websites without a risk of penalty.

### Recommendations to kratom vendors

In the long term, as emerging literature increasingly examines kratom’s potential risks and benefits, kratom consumers should have access to up-to-date information. There is currently insufficient research in the medical and scientific literature to substantiate such claims of kratom’s risks and benefits. Unfortunately, the current state of regulatory affairs and research leaves kratom consumers without the adequate information needed to make informed health decisions. Thus, in the meantime, kratom vendors should focus on utilizing structure–function claims to describe potential kratom benefits alongside the disclaimer statement explicated by the DSHEA.

Moreover, the vast majority of the websites examined in this study lack hyperlinks to quality external resources for further reading or research. Indeed, one way to improve DISCERN scores would be for kratom vendors to link and provide access to credible evidence-based sources that provide unbiased and balanced information concerning kratom (i.e., to improve on questions 4, 6, and 7). Secondly, given that kratom literature is rapidly evolving, such information should include the date it was published or updated (i.e., to improve on question 5). While many vendors have entire blogs dedicated to information about kratom (e.g., different strains, consumption methods, etc.), the consumer is left sorting through blog posts to find the desired information. This key information would ideally be displayed in a more consolidated, easy-to-read manner for consumers.

Additionally, to score more highly on DISCERN, online vendors could provide consumers with a broad level of understanding of how kratom works to elicit the described structure–function claims that are stipulated by DSHEA (i.e., to improve on question 9). For instance, websites can, to the extent they are able, discuss what is currently known – in lay terms – about mitragynine and 7-hydroxymitragynine, two active constituents that are known to interact with adrenergic, serotonergic, dopaminergic, and opioid receptors [30, 31]. However, it should be noted that (a) not all of the pharmacology of kratom is centered on these two primary alkaloids, (b) no human studies have been conducted to examine these mechanisms, and (c) kratom is a complex botanical that cannot be simplified by merely categorizing it in traditional categories such as “opioid” or “stimulant” [32, 33]. Presenting this uncertainty to consumers can also help improve DISCERN scores (i.e., to improve on question 8). Lastly, vendors could be clearer about potential risks and harms that have been described by those who use kratom on surveys, in public venues, and in case reports (i.e., to improve on question 11). Many websites provide minimal risk information for products or, more often, simply provide a generic disclaimer at the very bottom of the website which a consumer would only see if they scrolled down. Included in risk or disclaimer information should be how researchers and physicians are not aware of (a) the exact conditions which may benefit from kratom, if any, (b) do not fully understand what dose, frequency, and route of administration is effective or safe, and (c) do not know what drug-drug interactions may be riskier than others if one engages in polyuse. This is especially important as the method of consumption may impact the risk of developing tolerance or addiction to kratom [19].

### Study limitations

The current study faces several limitations. First, these websites are dynamic entities, and here the authors analyzed the content over a limited time frame. Websites may change rapidly upon new policies or vendor investment and interest. As such, the current DISCERN scores only reflect the time period utilized in the study. Second, while DISCERN has been shown to be a reliable and valid instrument for evaluating consumer health data, it is still open to user perceptions. To help mitigate this potential bias, our team was multidisciplinary with reviewers having varied backgrounds.

### Future studies

Further research related to online kratom vendors and consumer health information may consider a content analysis exploring a website’s disclaimer, blog posts, reviews from previous consumers, and more. Of particular interest may be how vendors describe the benefits of kratom under the current FDA limitations. Additionally, researchers may consider performing qualitative studies related to vendor perceptions and/or consumer perceptions about online kratom access. Lastly, it will be important to know the prevalence of blatant misinformation about kratom or kratom’s effects provided to consumers on vendor websites. However, there are many sources’ online consumers may use to find information about kratom, including Reddit or Facebook, making it difficult to ascertain exactly what information is or is not readily available to consumers [13].

## Conclusion

The health information provided to customers on kratom vendors’ websites is poor and lacks sufficient details regarding the benefits, risks, and uncertainties. Consumers are hence not provided with critical information required to make informed decisions about the use of kratom. Although the DISCERN scores were better for our study of the AKA GMP-qualified kratom vendors compared to the previous study of Canadian kratom vendors by Ng et al., there is considerable room for improvement. Such improvements can, and should, be supported by regulatory requirements and policies guided by both the AKA and legislative bodies. Lastly, clinicians should be keenly aware of the information available – or lack thereof – to those who use kratom, in order to have informed discussions through patient education.

## Data Availability

Data sharing is not applicable to this article as no datasets were generated or analyzed during the current study.

## Abbreviations

AKA: American Kratom Association
FDA: Food and Drug Administration
GMP: Good Manufacturing Practices
KCPA: Kratom Consumer Protection Act
DSHEA: Dietary Supplement Health and Education Act
